# Don’t be late! Postponing cognitive decline and preventing early unemployment in people with multiple sclerosis: a study protocol

**DOI:** 10.1186/s12883-023-03513-y

**Published:** 2024-01-15

**Authors:** Jip Aarts, Shalina R. D. Saddal, Judith E. Bosmans, Vincent de Groot, Brigit A. de Jong, Martin Klein, Marit F. L. Ruitenberg, Frederieke G. Schaafsma, Esther C. F. Schippers, Menno M. Schoonheim, Bernard M. J. Uitdehaag, Sabina van der Veen, Pauline T. Waskowiak, Guy A. M. Widdershoven, Karin van der Hiele, Hanneke E. Hulst, on behalf of the Don’t be late! consortium, Bram A. J. den Teuling, Pim van Oirschot, Sonja Cloosterma, Jos Vermeer, Chris C. Schouten, Gerard J. Stege, Thijs van ’t Hullenaar, Casper E. P. van Munster, Renske G. Wieberdink, Jolijn Kragt Judith Schouten, Erwin L. J. Hoogervorst, Paul A. D. Bouma, Floris G. C. M. De Kleermaeker, Meike Holleman, Sofie Geurts, Christaan de Brabander, Nynke F. Kalkers

**Affiliations:** 1 Health, Medical and Neuropsychology Unit, Institute of Psychology, Faculty of Social Sciences, Leiden University, Wassenaarseweg 52, Leiden, 2333 AK The Netherlands; 2 Leiden Institute for Brain and Cognition, Leiden, The Netherlands; 3 MS Center Amsterdam, Anatomy and Neurosciences, Vrije Universiteit Amsterdam, Amsterdam Neuroscience, Amsterdam UMC Location VUmc, Amsterdam, The Netherlands; 4 MS Center Amsterdam, Public and Occupational Health, Vrije Universiteit Amsterdam, Amsterdam UMC Location VUmc, Amsterdam, The Netherlands; 5 Department of Health Sciences, Faculty of Science, Vrije Universiteit Amsterdam, Amsterdam Public Health Research Institute, Amsterdam, The Netherlands; 6 MS Center Amsterdam, Rehabilitation Medicine, Vrije Universiteit Amsterdam, Amsterdam UMC Location VUmc, Amsterdam, The Netherlands; 7 MS Center Amsterdam, Neurology, Vrije Universiteit Amsterdam, Amsterdam Neuroscience, Amsterdam UMC Location VUmc, Amsterdam, The Netherlands; 8 Medical Psychology, MS Center Amsterdam, Vrije Universiteit Amsterdam, Amsterdam Neuroscience, Amsterdam UMC Location VUmc, Amsterdam, The Netherlands; 9 Ethics, Law & Medical Humanities, Vrije Universiteit Amsterdam, Amsterdam UMC Location VUmc, Amsterdam, The Netherlands

**Keywords:** Multiple sclerosis, Cognition, Exercise, Employment, Prevention, Health-related quality of life

## Abstract

**Background:**

Up to 65% of people with multiple sclerosis (PwMS) develop cognitive deficits, which hampers their ability to work, participating in day-to-day life and ultimately reducing quality of life (QoL). Early cognitive symptoms are often less tangible to PwMS and their direct environment and are noticed only when symptoms and work functioning problems become more advanced, i.e., when (brain) damage is already advanced. Treatment of symptoms at a late stage can lead to cognitive impairment and unemployment, highlighting the need for preventative interventions in PwMS.

**Aims:**

This study aims to evaluate the (cost-) effectiveness of two innovative preventative interventions, aimed at postponing cognitive decline and work functioning problems, compared to enhanced usual care in improving health-related QoL (HRQoL).

**Methods:**

Randomised controlled trial including 270 PwMS with mild cognitive impairment, who have paid employment ≥ 12 h per week and are able to participate in physical exercise (Expanded Disability Status Scale < 6.0). Participants are randomised across three study arms: 1) ‘strengthening the brain’ – a lifestyle intervention combining personal fitness, mental coaching, dietary advice, and cognitive training; 2) ‘strengthening the mind’ – a work-focused intervention combining the capability approach and the participatory approach in one-on-one coaching by trained work coaches who have MS themselves; 3) Control group—receiving general information about cognitive impairment in MS and receiving care as usual. Intervention duration is four months, with short-term and long-term follow-up measurements at 10 and 16 months, respectively. The primary outcome measure of the Don’t be late! intervention study will be HRQoL as measured with the 36-item Short Form. Secondary outcomes include cognition, work related outcomes, physical functioning, structural and functional brain changes, psychological functioning, and societal costs. Semi-structured interviews and focus groups with stakeholders will be organised to qualitatively reflect on the process and outcome of the interventions.

**Discussion:**

This study seeks to prevent (further) cognitive decline and job loss due to MS by introducing tailor-made interventions at an early stage of cognitive symptoms, thereby maintaining or improving HRQoL. Qualitative analyses will be performed to allow successful implementation into clinical practice.

**Trial registration:**

Retrospectively registered at ClinicalTrials.gov with reference number NCT06068582 on 10 October 2023.

## Background

Multiple sclerosis (MS) is a chronic, neurodegenerative, and demyelinating disease of the central nervous system [1]. Globally, the disease counts around 2.5 million cases. MS is usually diagnosed when people are between 20 and 40 years old, making it the most prevalent cause of disability in young adults of working age [2, 3]. MS causes a wide variety of symptoms, with fatigue, cognitive, and motor problems commonly reported [4]. Up to 65% of people with MS (PwMS) develop cognitive deficits [5, 6], which severely affect daily life functioning and ultimately health-related quality of life (HRQoL) [7]. About 65% of all PwMS become unemployed within 5 years after diagnosis [8–10], with cognitive impairment being one of the main reasons for unemployment and work-related problems [11, 12].

### MS and cognitive rehabilitation

Current rehabilitation for cognitive impairment in PwMS is limited and focusses mostly on restorative and compensatory strategies [13]. Previous studies consistently demonstrate mild-to-moderate effects of cognitive training on cognitive performance in PwMS (i.e., functional training [14]). A recent study suggests that people with relapsing remitting MS (RRMS) and larger grey matter volume were more likely to improve on information processing speed after cognitive training compared to people with progressive MS (PMS) and grey matter atrophy [15]. Furthermore, another study provides similar results in that there might be an early window of opportunity for cognitive training as PwMS with an intact brain network (compared to healthy controls) benefited from a cognitive rehabilitation programme, while PwMS with brain network deficits did not show beneficial effects from the intervention [16]. Next to cognitive training, earlier work has also shown that physical exercise appears to improve cognitive functioning in PwMS [17, 18]. Studies have shown that both cognitive training and exercise positively influence brain functioning [19–22]. Enhanced effects can be expected from the combination of cognitive training and exercise, as was illustrated in patients with mild cognitive impairment and Alzheimer’s disease [23]. However, the actual effects of such a combination on cognition still need to be established in PwMS.

### MS and work

With respect to work-related problems, interventions are typically only provided when PwMS are already on sick leave or have lost their job [24]. These interventions might therefore be too late, as having and keeping a job is important for people’s social contacts, self-respect and to feel valued [25]. In addition, job and/or productivity loss have economic consequences for both the PwMS as well as society at large [26]. For instance, already for the mildly affected MS group (expanded disability status scale score (EDSS) 0–3), the mean utility (i.e., value for given states of health between 1 (full health) and 0 (death)) and annual MS-related healthcare costs were estimated at 0.744 and €23,100 in the Netherlands respectively, which primarily resulted from productivity losses [27]. Research on work-related interventions for PwMS is directly needed but remains scarce. In fact, in recent years the ability to work and being employed increasingly received attention within healthcare research. This is not surprising as work participation is a significant determinant of HRQoL in PwMS, independent of their experienced health [28]. The Dutch government encourage individuals with a chronic disease such as MS to self-manage and take control of their lives, including their work [29]. However, self-managing daily demands in a dynamic work context where activities request a high level of cognitive and psychological skills is challenging. For PwMS, being able to work is therefore not only a matter of self-management of work challenges but is also highly dependent on the work context. A supportive work environment that is willing to adjust and fine-tune the work to the cognitive abilities of the employee and to provide emotional support is imperative for workers with chronic diseases such as MS to be able to remain employed [30]. As such, a proactive and timely work-related intervention that includes active involvement of the workplace may enable patients to effectively deal with work challenges and prevent sick leave and job loss.

### Don’t be late!

While the physical limitations of MS can (partly) be compensated with mobility aids (e.g., wheelchair, orthoses) and workplace adjustments, such solutions are scarce for cognitive deficits. Currently, interventions for cognitive impairments and work-related problems start when these problems are often already too advanced and difficult to overcome [25]. This triggers a negative cascade of events that inevitably leads to further cognitive deterioration, unemployment and decreased HRQoL. Therefore, it is of great importance to intervene in the early stage of cognitive impairment.

The Don’t be Late! project aims to provide timely intervention in PwMS with mild cognitive impairment who are still employed. The primary aim is to investigate the effectiveness of two innovative interventions as compared to enhanced usual care in improving HRQoL. These interventions are aimed at preventing and/or postponing cognitive decline and work-related problems. Secondary aims are, 1) to assess the effectiveness of the investigated interventions in improving cognitive, psychological and work functioning, and in enhancing the brain’s functional network, 2) to examine which factors (i.e., baseline cognitive, psychological, work and brain MRI-parameters) are predictive of the response to the investigated interventions, 3) to assess which mechanisms mediate the effect of the investigated interventions on HRQoL, and 4) to assess the cost-effectiveness of the investigated interventions.

For the qualitative study, the primary aim is to qualitatively reflect on the process and outcome of the investigated interventions considering the perspectives of relevant stakeholders and to investigate how to foster smooth and successful implementation in clinical practice.

## Methods/design

### Study design and setting

The Don’t be late! research project consists of three work packages, of which this protocol describes the second and third.

Work package 1—‘Timely identification of cognitive decline in Multiple Sclerosis’ aims to 1) identify early cognitive decline in PwMS, and 2) to validate the Multiple Screener in 750 PwMS, a digital tool for administering neuropsychological tests in PwMS [31].

Work package 2 concerns a randomised controlled trial containing two intervention arms (‘strengthening the brain’ and ‘strengthening the mind’) and a control condition (‘enhanced usual care’). This study follows a repeated-measures design and is performed at Amsterdam UMC and Leiden University, the Netherlands. Participants will be selected from a pool of eligible participants from work package 1 and participants will be recruited through other studies in which they indicated that they could be approached for further research participation, as well as through social media channels. Eligible individuals will be randomised over the three arms. Results of measurements for work package 2 overlapping with work package 1 will be adapted from work package 1 for participants included through work package 1.

Work package 3 is a qualitative study using semi-structured interviews with representatives from all stakeholder groups to investigate the process of the interventions. Additionally, focus groups are used to provide a deeper understanding of the results of the interventions and to investigate how to successfully implement the interventions into clinical practice.

### Study population

#### Work package 2: Randomized controlled trial

We aim to include 270 participants in the randomized controlled trial. In order to be eligible to participate, PwMS must meet the following criteria: (1) confirmed MS diagnosis according to the McDonald 2017 criteria [32], (2) age between 18 and 67, (3) no changes in disease modifying therapy in the last three months prior to inclusion—this criterion only applies at inclusion to ensure participants are in a stable situation at the start of the study and for follow-up measures, changes in treatment will be registered but will not result in exclusion from the study, (4) no current relapse or steroid treatment in the six weeks prior to study visits, (5) presence of mild cognitive deficits (at least one test with a Z-score of -1.0 to -1.99 below norm scores of healthy controls on the Minimal Assessment of Cognitive Function in Multiple Sclerosis (MACFIMS) battery [33]; not fulfilling the criteria for severe cognitive impairment (Z-score of -2.0 on ≥ 2 tests)), (6) performing paid work for at least 12 h per week, (7) being able to participate in an exercise intervention (i.e., EDSS < 6.0), and (8) fulfilling safety criteria for MRI (no metal inside body, not pregnant, no claustrophobia). Exclusion criteria are (1) presence of neurological (other than MS) and psychiatric disorders, (2) a current or history of drug or alcohol abuse, (3) being unable to speak or read Dutch, (4) currently on sick leave for a period of 6 weeks or longer, and (5) currently pregnant.

### Interventions

Participants will be randomly allocated into one of three groups, ‘*strengthening the brain’*, ‘*strengthening the mind’*, or ‘*enhanced usual care’*. The interventions ‘strengthening the brain’ and ‘strengthening the mind’ have a duration of four months and will be optimised towards a participant’s personal needs. The enhanced usual care group is acting as control group where participants are asked to continue life ‘as is’ for the duration of the intervention (4 months).

#### Strengthening the brain

‘Strengthening the brain’ is a lifestyle intervention which combines physical exercise with cognitive training.

For the exercise component, participants receive exercise and lifestyle coaching (part in kind contribution of Personal Fitness Nederland, Fit for Life programme, www.personalfitnessnederland.nl) where one-on-one training will be provided at one of the 94 studios of Personal Fitness Nederland (PFN). Every session will exist of a combination of cardio (aerobic) and strength (anaerobic) training dependent on the goals of the participant. At the start of the programme an intake will take place, where the physical fitness of the participant will be determined, and attention will be paid to specific health issues and goals of the participant. Sessions will be made increasingly more challenging. Each session will start with weighing the participant followed by 30 min of exercise in a studio where no other people are present, guaranteeing full attention to the participant. Next to the weekly face-to-face sessions, participants are asked to perform pre-set exercises at home twice a week (20 min each) which are guided by instruction videos on an online platform. A quantitative assessment of the adherence (how many sessions attended and progress between training sessions) will be done by the lifestyle coach and participants will write down their goals concerning exercise and diet for the upcoming week in a personal log. Lifestyle coaches will also provide participants with diet schemes and mental coaching. This will also be recorded using the personal log.

For the cognitive component, participants will follow cognitive training using a Dutch home-based computerised cognitive training, BrainGymmer^©^ (https://www.Braingymmer.com), which has been used in multiple studies [34–37]. A variety of cognitive functions are trained (information processing speed, spatial memory, working memory, executive functioning) rather than one cognitive function in particular. The cognitive training will focus on the cognitive function that was most impaired on the neuropsychological assessment at baseline. The training has an adaptive mechanism, which will adjust to the participant’s performance level to make it challenging for all participants. The programme will log training time and the percentage of correctly performed games. Participants are instructed to train for 60 min per week.

#### Strengthening the mind

The ‘strengthening the mind’ intervention consists of biweekly contact with trained work coaches who are all diagnosed with MS themselves (in kind contribution of the Dutch MS Society, MSVN). The intervention focuses on (re)discovery of a sustainable and healthy balance between relevant work values, the challenges workers with MS are facing, and at the same time meeting the work demands in a dynamic work context with ongoing technological developments. This will be reached using a combination of the capability approach and the participatory approach: “Working Positively”. The starting point of the capability approach related to work participation is to explore what people find important and valuable in work – what they would like to achieve in a given (work) context – and moreover, in the case of individuals confronted with a chronic and progressive disease such as MS, to ascertain whether people are enabled and able to do so. The participatory approach uses a practical, stepwise manner to detect challenges at the workplace and implement solutions by actively involving both the worker and the workplace (e.g., the supervisor).

Every participant will be matched with a trained work coach that also has an MS diagnosis. The starting point (step 1) of the coaching will be an assessment of individual work values using the capability set for work questionnaire [38] and to become acquainted with the worker and their working context. This starts by identifying which work values are important to the worker with MS. Secondly, it will be assessed to what extent workers are enabled to achieve these values at work, and thirdly to what extent they are able to achieve these values at work. Any discrepancies patients experience in being enabled and able to achieve important work values will be flagged, as these indicate barriers for optimal and satisfactory work participation [39]. Additionally, the employer or other representative of the workplace will be requested to join at least one coaching session to provide information related to work demands and context from their perspective as is described as an essential element of the participatory approach. In case the participant prefers not to disclose their MS diagnosis to the workplace, an independent occupational health professional may act as a representative of the workplace.

In the second step of this intervention, both the worker and the representative of the workplace prioritise the important values and challenges to meet work demands (e.g., work tasks, time pressure, working hours, peak loads, or other challenges within the working context). The worker and representative of the workplace will select three work challenges that will be addressed during the coaching period. Third, for each work challenge both the worker and the representative of the workplace will think of possible and practical solutions under guidance of the coach. Fourth, a plan of action is developed in which consensus for the proposed solutions are achieved and a plan for its execution is agreed upon. The fifth step describes and ensures the implementation of solutions, and in the final step the degree and the effects of the implemented solutions will be evaluated with all stakeholders involved. If necessary, the plan of action is adjusted [40]. The intervention is completed either when satisfactory solutions have been implemented for all identified work challenges, or after biweekly coaching has taken place for a period of 4 months.

#### Enhanced usual care

Participants in the enhanced usual care condition will watch a pre-recorded video together with a researcher with the opportunity to ask questions afterwards. The video provides a standardised explanation of cognitive decline in PwMS based on the Dutch book “MS and Cognition, by scientists for people with MS and their surroundings” (editors: Hanneke Hulst & Jeroen Geurts). The video includes information about the frequently affected cognitive domains in MS and their relation to brain pathology. Participants will be asked to continue their life ‘as is’ during the time of the intervention (4 months). The main reason for incorporating enhanced usual care in the protocol is to avoid resentful demoralisation of participants assigned to this group.

### Outcome measures

#### Work package 2: Randomized controlled trial

**Fig. 1:**
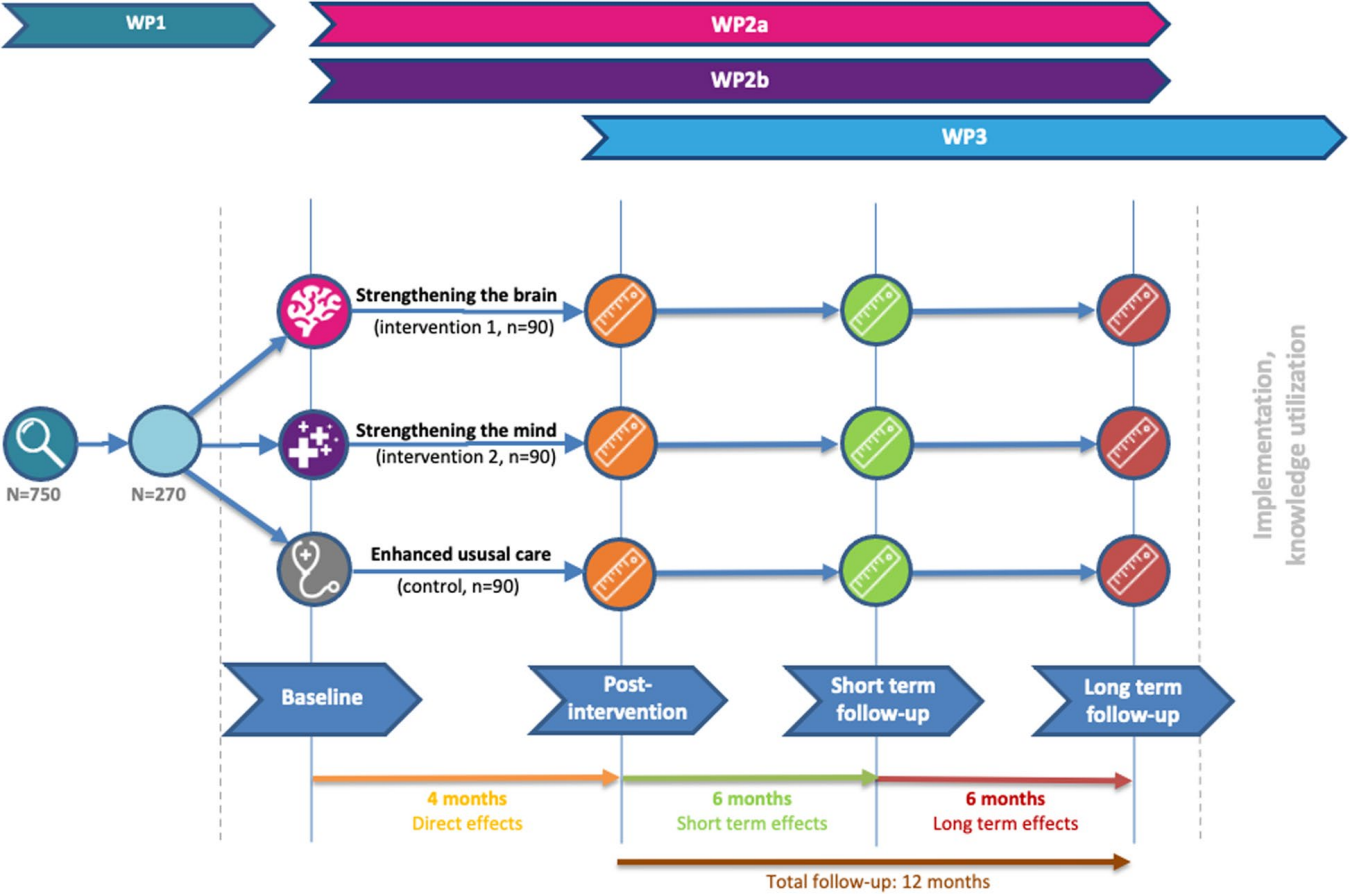
Participant timeline of Don’t be late! project. WP = work package

**Fig. 2:**
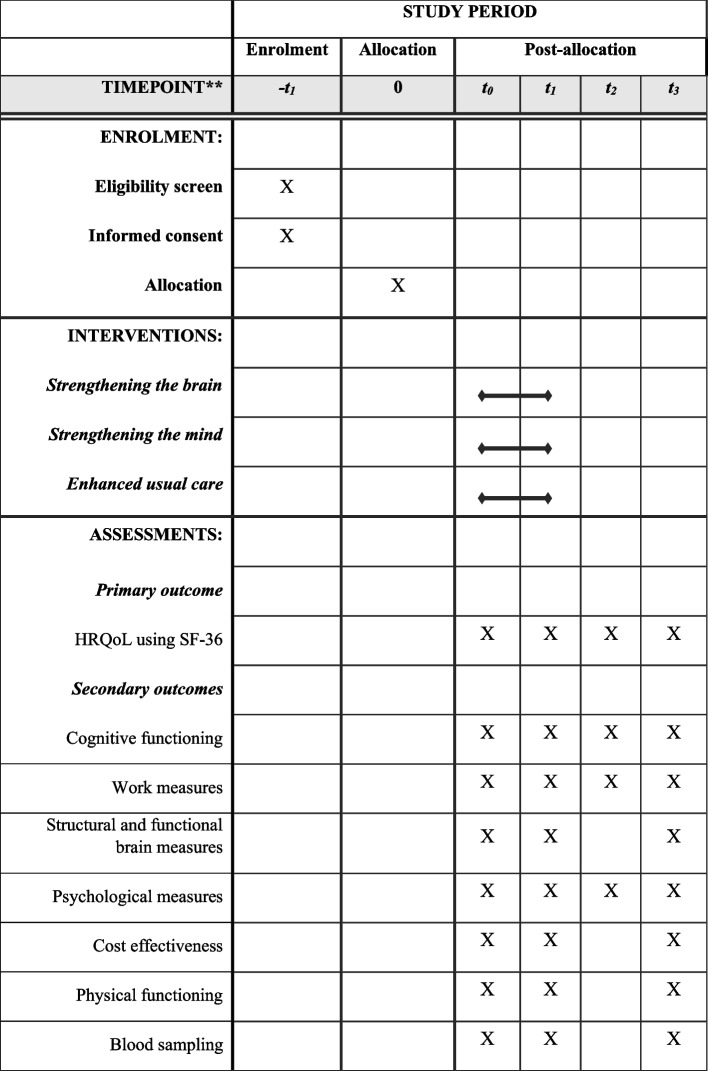
Time points of all assessments. t0 = baseline, t1 = month 4, directly after intervention, t2 = month 10, short-term follow-up, t3 = month 16, long-term follow-up

As illustrated in Figs. 1 and 2, measurements in the randomized controlled trial will take place right before the intervention (baseline; T0), directly after the intervention (four months after baseline; T1), at short- and long-term follow-up (10 months (T2) and 16 months (T3), respectively).

#### Demographic and disease-related measures

During the baseline assessment, demographic and clinical characteristics will be gathered from the participants. The following characteristics will be determined: Age, sex, length, weight, highest level of education attained, job type, working hours, disability pension, current and history of exercise activity, year of diagnosis, MS subtype, disease duration, disease severity using EDSS, and medication history.

#### Primary outcome measure

The primary outcome measure of this study is HRQoL, which will be assessed using a composite score of the 36-item Short Form (SF-36) [41]. HRQoL will be determined at all four measurement moments during the study, which will allow to assess short-term and long-term changes in HRQoL. The effects of the interventions on the SF-36 as overall value and per subcategory will be analysed for timepoints T1, T2, and T3. Subcategories of the SF-36 contain physical functioning, role limitations because of physical health problems, bodily pain, social functioning, general mental health, role limitations because of emotional problems, vitality, and general health perceptions. The validity and reliability of the SF-36 are well established in healthy controls and PwMS [41, 42].

#### Secondary outcome measures

##### Cognitive measures

Cognitive functioning will be assessed using the MACFIMS battery [33] and the Multiple Sclerosis Neuropsychological Screening Questionnaire (MSNQ) [43]. The MACFIMS battery consists of the following tests: Dutch adaptation of the Controlled Oral Word Association Test (COWAT); Dutch Letter Fluency Test [44, 45], Judgement of Line Orientation (JLO) [45], Dutch version of the California Verbal Learning Test, second edition (CVLT-II) [46–48], Brief Visuospatial Memory Test-Revised (BVMT-R) [49], Paced Auditory Serial Addition Test (PASAT) [50], Symbol Digit Modalities Test (SDMT) [51], and the Sorting Test from the Delis-Kaplan Executive Function System (DKEFS) [52]. The SDMT and PASAT include the adaptations from Rao [53]. To test for performance validity, the Amsterdam Short Term Memory Test (ASTM) [54] and the Rey 15-Item Test [55] will be assessed. The Rey 15-Item Test will only be assessed if the total score on the ASTM indicates underperformance (a cut-off of ≤ 84 will be applied).

##### Work measures

Measures reflecting on work include work participation and productivity, assessed using the Work Productivity and Activity Impairment Questionnaire: General Health [56], work difficulties, using the Multiple Sclerosis Work Difficulties Questionnaire [57, 58], the capability to carry out work activities, assessed with the Capability Set for Work Questionnaire [39] which has been used in previous studies with workers with MS [38], and quality of working life, which will be assessed with the valid and reliable Quality of Working Life Questionnaire for Cancer Survivors [59] which has been used in multiple patient populations [60–62].

##### Structural and functional brain measures

The MRI scan features an expanded clinical protocol, focused on brain and lesion volumes and structural and functional connectivity. Lesion masks and volumes will automatically be detected on 3D-FLAIR [63]. Grey matter volume, white matter volume and total brain volume will be determined using FSL-SIENAX, after lesion filling [64] on the 3DT1. Volumes of the deep grey matter structures will be determined using FIRST [65], which will also be subtracted from SIENAX-derived segmentations to derive total cortical volume. Cortical thickness will be determined using Freesurfer (Charlestown, Massachusetts).

Diffusion Tensor Imaging (DTI) will be performed to investigate the microstructural integrity of the white matter [66]. Tract Based Spatial Statistics (TBSS, FSL) will be used to investigate structural integrity across the main white matter tracts in the brain [67]. Furthermore, probabilistic tractography using MRTrix will be used to visualise specific tracts in the white matter to determine structural connectivity and the volume of the specific white matter tracts of interest [68–70].

The amplitude of regional functional activation will be determined using blood-oxygen level dependent (BOLD) response during an episodic memory encoding task. The task has been specifically developed to assess memory function and robustly evokes brain activation in the hippocampus [71, 72], showing hippocampal changes in MS. It makes use of an event-related design of which only the correctly remembered items will be modelled (to ensure proper attention). The task contains different landscapes which have to be judged to be tropical landscapes or non-tropical landscapes. A retrieval task will be held after the MRI scanning has finished. FSL-FEAT will be used to analyse the BOLD responses for the correctly remembered items.

Resting-state fMRI will be used to assess functional connectivity (FC). Images will be pre-processed using FSL and corrected for motion using ICA-AROMA. FC will be calculated by correlating the averaged time series of brain regions. These regions are defined using the cortical Brainnetome atlas, and the deep grey matter atlas that is part of FIRST. Subsequently, all pair-wise connectivity scores will be corrected for the whole-brain mean, to deal with individual fingerprint effects [73]. Dynamic FC will be quantified by separating time series into sliding windows, calculating the variability over time of functional connectivity strength [74]. Static and dynamic FC patterns will be summarized across regions forming separate resting-state networks, such as the default-mode and fronto-parietal networks [75]. In addition, we will use connectivity patterns to calculate measures of static and dynamic network topology, such as global and local efficiency, using the Brain Connectivity Toolbox (BCT) in Matlab (Natick, Massachusetts: The MathWorks Inc.) [76]. Network topology will also be assessed using eigenvector centrality mapping, which determines the network importance of individual regions, which was previously validated for MS [77].

##### Psychological measures

Fatigue will be assessed using the Checklist Individual Strength (CIS) [78], validated in Dutch PwMS [79]. Mood and anxiety will be tested using the Hospital Anxiety and Depression Scale (HADS) [80] which has been validated in PwMS [81]. Resilience will be measured using the valid and reliable Connor Davidson Resilience Scale [82]. Perceived level of stress will be assessed using the Perceived Stress Scale (PSS) [83], validated in PwMS [84]. Social mindfulness will be assessed using the paradigm by van Doesum et al. (2013) [85], the standard assessment to measure social mindfulness. Social participation will be measured using the valid and reliable PROMIS ‘Ability to Participate in Social Roles and Activities’ item bank [86].

##### Societal costs and general quality of life

Societal costs include healthcare, patient and family, and lost productivity costs and will be assessed using the iMTA Productivity Cost Questionnaire (iPCQ) [87] and iMTA Medical Cost Questionnaire (iMCQ) [88]. Additionally, the EuroQol five-level questionnaire (EQ-5D-5L) will be used to measure general quality of life. The Dutch EQ-5D-5L tariff will be used to convert EQ-5D-5L health states to utility scores to enable the calculation of quality-adjusted life-years (QALYs) [89, 90].

##### Physical functioning

In order to examine physical functioning, balance, walking speed, endurance, grip strength, and dexterity will be assessed. Balance will be assessed using the Mini-BESTest, which has been shown to be a reliable and valid balance assessment in PwMS [91, 92]. The test contains 14 items which can be categorised into anticipatory postural adjustments, reactive postural control, sensory orientation, and dynamic gait. Each item can be scored on a 3-point scale. A total score of 28 points can be achieved, where a score < 19 induces an increased risk of falling [91]. Walking speed will be measured using the Timed 25-Foot Walk (T25FW), which is a valid and reliable measure to assess ambulatory performance [93]. Participants will walk between two cones, 7.62 m apart. Participants will perform the T25FW four times, two times as fast as possible and two times on their comfortable walking speed [94]. Endurance will be measured using the Shuttle Walk Test (SWT). It is a basic test which can be conducted with few materials. The SWT has recently been validated and is proven to be a reliable outcome measure in ambulatory PwMS [95]. For this study, the protocol of Singh et al. (1992) will be used [96].

Grip strength will be assessed using a JAMAR hand-held dynamometer and will be expressed in kilograms. Participants are asked to pinch the dynamometer in a seated position with their arm held out in a 90-degree angle two times with each hand [97]. Upper limb dexterity will be measured with the combination of the 9-Hole Peg Test (9HPT) [98] and the Purdue Pegboard Test (PPT) [99]. The 9HPT is an often-used measure for dexterity in PwMS [98]. To additionally assess bimanual motor function, the PPT is included. Grip strength, 9HPT, and PPT are valid measures for upper limb assessment in PwMS [100].

##### Blood sampling

Blood will be drawn at three timepoints and will be stored in a biobank created for this study such that markers of interest can be studied retrospectively.

##### Effectiveness and adherence of treatment protocol

A quantitative assessment of the ‘strengthening the brain’ and ‘strengthening the mind’ programmes will be conducted using the Goal Attainment Scaling (GAS) [101]. Participants will formulate, together with a researcher or work coach, three to four goals following the SMART (Specific, Measurable, Attainable, Realistic, Timely) principle. For each goal, the expected or ‘level 0’ outcome will be carefully defined at baseline. Goals will be weighted for importance and difficulty. At the end of the intervention, the participant and researcher or coach will agree upon if the level of the goal was achieved (0); slightly exceeded (+ 1) or greatly exceeded (+ 2); or if it was ‘not quite achieved’ (-1) or ‘nowhere near’ (-2). The lifestyle coach in the ‘strengthening the brain’ intervention will assess how many sessions were attended and will also assess the progress between training sessions. Similarly, adherence to the cognition training within ‘strengthening the brain’ will be logged (e.g., training time and percentage of correctly performed games). In the ‘strengthening the mind’ intervention, the work coach will note the number, duration, and form of the consults (face-to-face and/or online), the number of consults the workplace representative was involved in, the role of the workplace representative, the three identified work challenges, and the proposed solutions to these work challenges. The work coach will record for each challenge the worker indicated to what extent the challenge was successfully addressed at the end of the coaching period using the Visual Analogue Scale (VAS) ranging from 0 to 100%.

### Work Package 3: Qualitative study

A selection of stakeholders involved in the project (PwMS, lifestyle- and work coaches, neurologists, neuropsychologists, occupational physicians, and occupational therapists) will be invited for the semi-structured interviews and focus groups. The interviews aim to provide insight into the experiences of stakeholders regarding the interventions and will be planned in batches to ensure an equal number of participating patients and coaches during the whole study period, preferably within a timeframe of 3 months from the last training session to avoid recall-bias. Interviews will be based on a topic list to ensure that all relevant questions will be addressed, and will continue until saturation is reached, meaning that no new themes emerge from the analysis. In case logistical improvements are brought up during the early interviews, adjustments will be made for future participants (e.g., if participants prefer to exercise in the morning rather than in the evening). Content-wise no changes will be allowed to the interventions. It is expected that 12–15 interviews with participants and 10–12 interviews with professionals from each group will be sufficient to reach saturation. Focus groups will be organised to further understand the effects of the ‘strengthening the brain’ and ‘strengthening the mind’ interventions, and to explore factors that promote or hinder the implementation of the interventions. The focus groups will consist of 8–10 participants to ensure optimal exchange of perspectives and dialogue and a script will be prepared. First, we will organize homogeneous focus groups (with participants and professionals separately); next, a heterogeneous dialogue group will be held in which participants and professionals reflect on the experiences and outcomes together. In forming focus groups, we will include diverse participants (considering sex, age, educational level). Focus groups will be organised after the individual interviews have been conducted and analysed, and short-term quantitative measures have been assessed.

As summarised in Table 1, a total of nine focus groups will be organised. For each of the two interventions, three focus groups will be organised, one with PwMS (group 1 and 2), one with coaches (group 3 and 4), and one heterogeneous group (a combination of PwMS and coaches/supervisors, group 5 and 6). One focus group will be organised for the work supervisors for the intervention ‘strengthening the mind’ (group 7). To enable implementation of the interventions in the near future, we will also organise a focus group with (referring) health care professionals (neurologists, neuropsychologists, occupational physicians/therapists; group 8), and a mixed group of PwMS and work supervisors that participated in the interventions and coaches of both interventions and healthcare professionals to discuss what is needed to introduce the interventions in practice successfully (group 9). Interviews and focus groups will be audio-recorded and transcribed to be further analysed.

**Table 1:** Overview of focus groups

Group number	Role
1	Participants intervention ‘strengthening the brain’
2	Participants intervention ‘strengthening the mind’
3	Coaches ‘strengthening the brain’
4	Coaches ‘strengthening the mind’
5	Heterogenous group participants & coaches ‘strengthening the brain’
6	Heterogenous group participants & coaches ‘strengthening the mind’
7	Work supervisors ‘strengthening the mind’
8	Health care professionals
9	Mixed group, participants, coaches & healthcare professionals

### Sample size

The sample size of 270 participants is based on a power calculation. A review of earlier studies on the effects of cognitive rehabilitation in MS [14] suggests that we can expect moderate effects (effect size of 0.35) between pre- and post-intervention. Assuming statistical significance of 0.025, a power of 0.80, and an effect size of 0.35, 75 participants per group are needed. A conservative alpha of 0.025 has been chosen to take into account that we compare two interventions with a control group. A correlation of 0.6 between the measures was assumed. Based on previous experiences, a drop-out of 20% over the study period is expected. Therefore, we will include 90 subjects per group to ensure sufficient power. This study will be carried out using an intention-to-treat protocol. Therefore, subjects who withdraw from the study after inclusion will not be replaced.

### Recruitment

Most of the participants participated in work package 1 before participating in the current study. These participants will mainly be recruited through 12 hospitals in the Netherlands, each having an MS population of 250–650 people. Of the 750 participants who take part in work package 1, we expect that approximately 30% has mild cognitive deficits [102], and therefore 220 participants who can enrol in the current study. The remaining 50 participants will be recruited through social media, and we can approach potential participants for this study by contacting PwMS who gave permission to be approached for further research projects. PwMS who are willing to participate will be checked for eligibility based on the in/exclusion criteria. People who are eligible to participate in work packages 2 and 3 will receive an information letter and will be asked to sign informed consent.

### Allocation and blinding

After baseline measurements, participants will be randomly assigned to one of the three conditions with a 1:1:1 allocation using a block randomisation of Research Randomizer (https://www.randomizer.org) to ensure equal group sizes. The block randomisation will not be disclosed to researchers performing measurements and analyses. After receiving informed consent, the involved lifestyle coach or work coach will be informed of the inclusion of the participant in their treatment arm by a researcher who is not involved with the measurements and analyses. Cognitive measures, structural and functional brain measures, neurological-, blood-, and physiological measures will be collected, analysed, and stored under a single-blinded protocol. To achieve single blinding, eligible participants will be randomised and assigned to an intervention by three designated researchers (SV, MR, KH) who are not involved in data collection and analyses. The researchers who are involved in data collection and analyses (JA, SS) are not informed about the allocation, nor discuss the intervention with the participants and involved coaches. In addition, researchers who carry out the measurements will explicitly instruct participants not to disclose any information about the intervention they are following.

### Data capture and data monitoring

Before the start of recruitment, study researchers responsible for data collection are trained on the use of assessments. All study data, except the structural and functional MRI data, are stored in Castor Electronic Data Capture (EDC) system, which is a secure, web-based application with features like audit-trails, monitoring, and capturing and integrating external data. Additionally, regular monitoring will be carried out through the sponsor to ensure data quality, accuracy, and GCP adherence. Collected data will be stored using a code and will always be checked by a second researcher to minimise input errors. Imaging data will be stored on the image data server of the hospital.

### Statistical analysis

Data will be analysed using R (R Core Team, Vienna, Austria) and Rstudio version 4.2 or higher (PBC, Boston, MA) and/or SPSS version 28 or higher (IBM, Armonk, NY). Analyses will be performed according to intention-to-treat and per-protocol. The main focus of the analyses will be on the intention-to-treat analysis, as this will reduce bias and better represents daily practice. A per-protocol analysis will be performed to evaluate the effect of the intervention on itself, supplementary to the intention-to-treat analysis. Data will be included in the per-protocol analysis if the participant completed the intervention. The alpha level will be set at a statistical threshold of α = 0.05, corrected for multiple comparisons when applicable. GAS will be used to discriminate between responders and non-responders concerning the interventions.

For the outcome cognitive functioning we will calculate a reliable change index (RCI) for each cognitive test based on the enhanced usual care group. Using the RCI scores allows us to correct for learning effects. An RCI score above + 1.64 or below -1.64 is assumed to reflect significant improvement or decline, respectively [5, 103]. The psychological and work functioning data will be obtained via questionnaires and will be analysed as continuous variables together with the physiological data. MRI images will be analysed using FSL (https://fsl.fmrib.ox.ac.uk) and Freesurfer (Charlestown, Massachusetts). Data obtained (atrophy, white matter integrity, task-specific brain activation and brain connectivity) will be exported to Rstudio after which mixed-model analyses will be performed.

Missing data will be minimised by using digital questionnaires, that prompt participants to answer each question before being allowed to proceed. For other outcome measures, multiple imputation will be used for missing baseline and follow-up data. Outliers will be identified and excluded from the main analysis.

Primary analyses will evaluate the effectiveness of the investigated interventions (‘strengthening the brain’, ‘strengthening the mind’) compared to a control group (‘enhanced usual care’) in improving HRQoL (overall score on SF-36). A mixed-model analysis will be performed, with time (T0, T1, T2, and T3) and condition (‘strengthening the brain’, ‘strengthening the mind’, and ‘enhanced usual care’) as fixed factors, and subject as random factor. P-values and estimates of effect sizes will be obtained. Additionally, separate mixed-model analyses will be performed to evaluate the effect of the investigated interventions compared to the control group on cognitive, psychological, physiological, and work functioning, and enhancing the brain’s functional network. Predictors of treatment response will be investigated by adding baseline scores on the biological, psychological, and environmental measures to the mixed-model analyses.

Mediation analyses will be used to study which mechanisms (i.e.., biological, psychological, and environmental) mediate the effect of the interventions on HRQoL. Conditions for mediation will be tested [104]: 1) investigated interventions should affect HRQoL; 2) investigated interventions should affect the presumed mediator; 3) presumed mediator and HRQoL should be related.

The cost-effectiveness analyses will be performed from a healthcare and a societal perspective according to the intention-to-treat principle. Intervention costs will be calculated using a bottom-up micro-costing approach. Missing cost and effect data will be imputed using multiple imputation by chained equations (MICE) with predictive mean matching to account for the skewed distribution of costs. The number of imputed datasets will be increased until the loss of efficiency is smaller than 5% [105]. Each dataset will be analysed separately as described below, after which results will be pooled using Rubin’s rules. Bivariate regression models will be used to estimate cost and effect differences between the intervention groups and the control group, while adjusting for confounders if necessary. Incremental cost-effectiveness ratios will be calculated by dividing the difference in costs by the difference in effects. Statistical uncertainty will be estimated using bias-corrected and accelerated bootstrapping and will be presented in cost-effectiveness planes and acceptability curves. Sensitivity analyses are performed to assess the robustness of the results.

#### Qualitative analysis

The analysis of the interviews will delve into the experiences of stakeholders with the interventions. The analysis of the focus groups focuses on interpretation of the effects and possible explanations of the interventions. Additionally, analysis of the focus groups explores factors that influence the implementation.

For the qualitative study, thematic analysis will be used to identify recurrent themes of meaning within the qualitative data [106]. After transcription, the data will be analysed and open coded using ATLAS.TI version 23 or higher. First the smallest units possible will be determined and coded, next the coded segments will be combined to identify themes.

### Current status

Participants are currently being recruited. The first participant was included on April 16th, 2023.

## Discussion

In this multi-arm, single-blind, controlled trial, 270 PwMS will be randomly assigned to a lifestyle intervention, a work intervention or enhanced usual care. Interventions will have a duration of four months with a total follow-up time of 12 months, which is a longer follow-up period than typical for these types of interventions and thus allows us to evaluate long-term effects. The interventions will be tailor-made depending on the individual needs of the participant.

In the two different interventions, HRQoL is enhanced via two hypothesized working mechanisms. In strengthening the brain we aim to postpone the development of cognitive decline through a combination of physical exercise, one-on-one mental coaching, dietary advice, and cognitive training. In strengthening the mind we aim to prevent job loss through combining the capability approach and the participatory approach in one-on-one coaching. In this study we specifically aim for PwMS with only mild cognitive impairment, thereby intervening when problems are not advanced yet and prevention may still be possible. The study focusses on HRQoL, which, along with other outcome measures, will be monitored for a total of 16 months, aiming to enhance our understanding of the effectiveness of the interventions over a longer period. Additionally, the involvement of a wide variety of specialised personnel reflects an interdisciplinary approach, resulting in a broad view on what is important for improving HRQoL in PwMS. The qualitative study adds insights from the participants’ and relevant stakeholders’ experiences during the interventions. This will enable interpretation of the found effects and provide insights into factors relevant for implementation into clinical practice.

The interventions ‘strengthening the brain’ and ‘strengthening the mind’ are aimed at two different problems: cognitive decline and job-loss. Both interventions use a tailor-made approach and are both aiming for an improvement in HRQoL albeit via different working mechanisms. Combining such diverse interventions might be a way forward to improve care for this group as problems are rarely one-sided. Additionally, the interventions are designed in co-design with end users to make it more feasible to adapt towards clinical practice.

In summary, the outcome of this study is expected to support the paradigm shift from symptom management towards preventative interventions, ultimately improving HRQoL in PwMS.

## Data Availability

No datasets were generated or analysed during the current study.

## Abbreviations

9HPT: 9-Hole Peg Test
ASTM: Amsterdam Short Term Memory Test
BCT: Brain Connectivity Toolbox
BOLD: Blood-Oxygen Level Dependent
BVMT-R: Brief Visuospatial Memory Test-Revised
CIS: Checklist Individual Strength
COWAT: Controlled Oral Word Association Test
CVLT-II: California Verbal Learning Test, second edition
DKEFS: Sorting Test from the Delis-Kaplan Executive Function System
DTI: Diffusion Tensor Imaging
EDC: Electronic Data Capture
EDSS: Expanded Disability Status Scale
EQ-5D-5L: EuroQol five-dimensional questionnaire
FC: Functional Connectivy
HADS: Hospital Anxiety and Depression Scale
HRQoL: Health-related quality of life
iMCQ: IMTA Medical Cost Questionnaire
iPCQ: IMTA Productivity Cost Questionnaire
JLO: Judgement of Line Orientation
MACFIMS: Minimal Assessment of Cognitive Functioning in Multiple Sclerosis
MS: Multiple Sclerosis
MSNQ: Multiple Sclerosis Neuropsychological Screening Questionnaire
MSVN: MS vereniging Nederland
PASAT: Paced Auditory Serial Addition Test
PFN: Personal Fitness Nederland
PMS: Progressive MS
PPT: Purdue Pegboard Test
PSS: Perceived level of Stress
PwMS: People with MS
QALYs: Quality-Adjusted-Life-Years
QoL: Quality of life
RCI: Reliable Change Index
RRMS: Relapsing remitting MS
SDMT: Symbol Digit Modalities Test
SWT: Shuttle Walk Test
T25FW: Timed 25-Foot Walk
TBSS: Tract Based Spatial Statistics
VAS: Visual Analogue Scale
